# Short-term inflow forecasting in a dam-regulated river in Southwest Norway using causal variational mode decomposition

**DOI:** 10.1038/s41598-023-34133-8

**Published:** 2023-04-29

**Authors:** Mojtaba Yousefi, Jinghao Wang, Øivind Fandrem Høivik, Jayaprakash Rajasekharan, August Hubert Wierling, Hossein Farahmand, Reza Arghandeh

**Affiliations:** 1 Department of Computer Science, Electrical Engineering and Mathematical Sciences, Western Norway University of Applied Science, Bergen, Norway; 2 Department of Electric Power Engineering, Norwegian University of Science and Technology, Trondheim, Norway; 3 Lyse Produksjon AS, Stavanger, Norway

**Keywords:** Hydrology, Engineering

## Abstract

Climate change affects patterns and uncertainties associated with river water regimes, which significantly impact hydropower generation and reservoir storage operation. Hence, reliable and accurate short-term inflow forecasting is vital to face climate effects better and improve hydropower scheduling performance. This paper proposes a Causal Variational Mode Decomposition (CVD) preprocessing framework for the inflow forecasting problem. CVD is a preprocessing feature selection framework that is built upon multiresolution analysis and causal inference. CVD can reduce computation time while increasing forecasting accuracy by down-selecting the most relevant features to the target value (inflow in a specific location). Moreover, the proposed CVD framework is a complementary step to any machine learning-based forecasting method as it is tested with four different forecasting algorithms in this paper. CVD is validated using actual data from a river system downstream of a hydropower reservoir in the southwest of Norway. The experimental results show that CVD-LSTM reduces forecasting error metric by almost 70% compared with a baseline (scenario 1) and reduces by 25% compared to an LSTM for the same composition of input data (scenario 4).

## Introduction

Inflow forecasting is an essential parameter for optimal water management and the operation of hydropower systems. However, human activities and climate change have made inflow forecasting an even more challenging problem in the face of intensifying flooding and drought events^1,2^. Furthermore, sudden decreases or increases in river flow negatively impact ecology, biodiversity, economy, wildlife, and human welfare. Hence, hydropower producers play an important role in mitigating such impacts in downstream river systems by storing or discharging water to rivers. For example, in Norway, hydropower producers must meet the government’s environmental requirements to guarantee a sustainable ecological state of rivers downstream^3^. Therefore, researchers have developed and investigated numerous inflow short-term forecasting models in recent decades to help hydropower operators and government agencies.

Machine learning-based models for inflow forecasting exhibited high performance in recent years, which is in line with the overall advancements in data science to solve complex and nonlinear problems. For example,^4–6^ used machine learning to identify functional relationships between inflow and other meteorological and hydrological variables. Deep learning methods with straightforward implementation are the prime movers for short-term inflow forecasting methods^7–10^. However, machine-learning methods are biased toward the training data and suffer from over-fitting, or under-fitting issues that limit the generalization and scalability in solutions^11^. Inflow forecasting as a multivariate and multi-domain (hydrology, meteorology, energy, etc.) problem even is more vulnerable to inherent biases in classic machine learning.

This paper uses two powerful tools from signal processing (Variational Mode Decomposition) and causal inference areas to create a novel pre-processing and feature extraction framework for multivariate and multi-domain time-series problems. Our method is called the causal variational mode decomposition (CVD). CVD discovers temporal and spatial dependencies among hydrological and meteorological parameters to find the most informative features regarding water inflow.

Variational mode decomposition (VMD) at the heart of CVD is a prominent Multi-resolution analysis (MRA) technique in the signal processing field, which is not based on wavelets to achieve higher performance with lower computing power^12,13^. Moreover, the step-wise sampling decomposition mechanism used in^14^ is employed for the VMD method to address the information leak problem from test and validation data to training data^15,16^. Further clarification is provided in the methodology section.

A significant shortfall for machine learning approaches in complex problems, including inflow forecasting, is the confusion between association and causation, which bottlenecks to selecting the most compelling features in a dataset with various cause-and-effect relationships. In addition, performing forecasting tasks with irrelevance or less relevant variables introduces uncertainty and bias into the outcomes and increases the computational time.

The main contributions of this paper are summarized as follows:

- This paper proposes a piggyback pre-processing framework to extract the most informative features related to a target variable among various input variables. CVD is an innovative integration between causal inference and the variational mode decomposition for multi-domain systems.
- Our proposed CVD framework is applied to the short-term inflow forecasting problem for a cascaded reservoir in the Western part of Norway using various hydrological and meteorological variables.

## Use case

### Location and facilities

The use case in this paper belongs to Lyse Produksjon AS, a company in Norway. Lyse has an annual production of 9.5 TWh of hydroelectric energy. Lyse relies on hourly inflow forecasting to optimize the inflow bypass from their upstream reservoir to reduce the ecological damages downstream per regulations by The Norwegian Energy Regulatory Authority (NVE)^17^.

**Figure 1:**
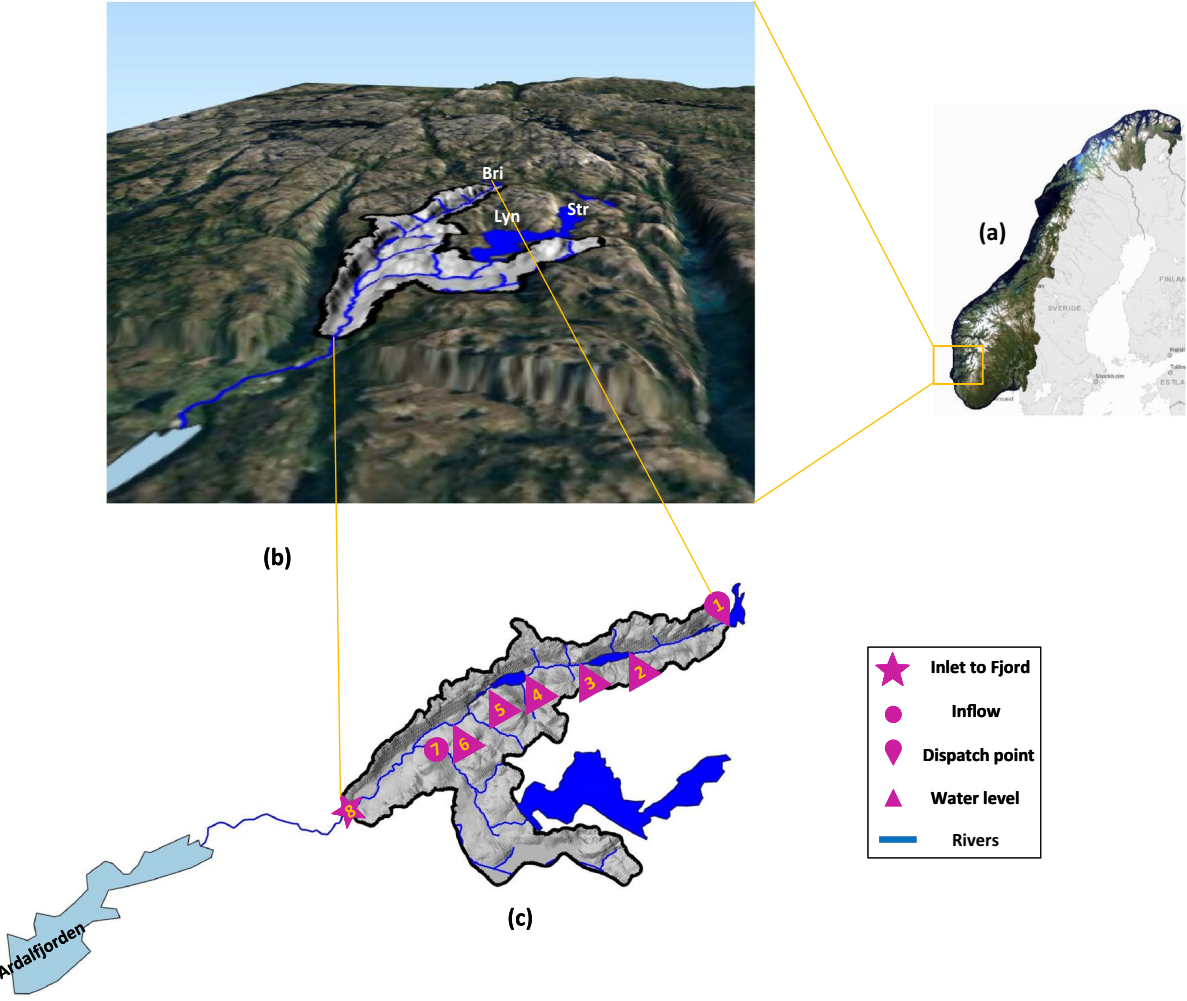
Study area overview which is located in Lyse Fjorden area in western Norway close to Stavanger: (**a**) location of the use case in Norway; (**b**) satellite view of the use case; (**c**) topography of the use case and measurement points. Note that the figure is generated by authors using QGIS 3.18 v 2022. (https://www.qgis.org/en/site/forusers/download.html).

The location of the use case is in Hjemland, Rogaland, southwest of Norway, close to Stavanger, see Fig. 1a. The Lyse has ownership interests in two main hydropower stations: Lyseboten I and II, together with three main reservoirs Breiavatnet (Bri), Lyngsvatnet (Lyn), and Strandvatnet (Str), see Fig. 1b. In this area, Lyse needs to manage the flow of the Stråna River by controlling the water dispatch from Bri reservoir (Location1) to downstream Kalltviet (Location8). The distance between Location1 and Location8 is nearly 20 (Km) where NVE measures inflow. As presented in Fig. 1c, along the river, there are four cascaded lakes: Mustdalsvatnet (Mus, Location2), viglesdasvatn (Vig, Location3 and Location4), Hiavatnet (Hia, Location5), and Hiafosen (Hio Location6) in which their water level and water temperatures are measured by Lyse. The measurement stations are presented with pink triangles, a circle, and a star.

### Collected data

**Figure 2:**
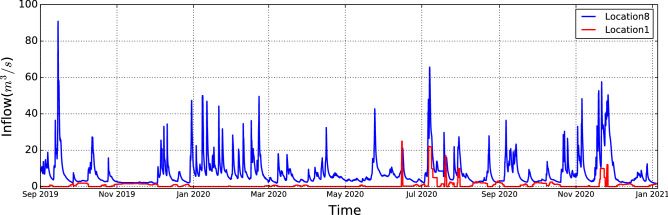
Downstream water inflow and upstream water dispatch from September 2019 to January 2021.

**Figure 3:**
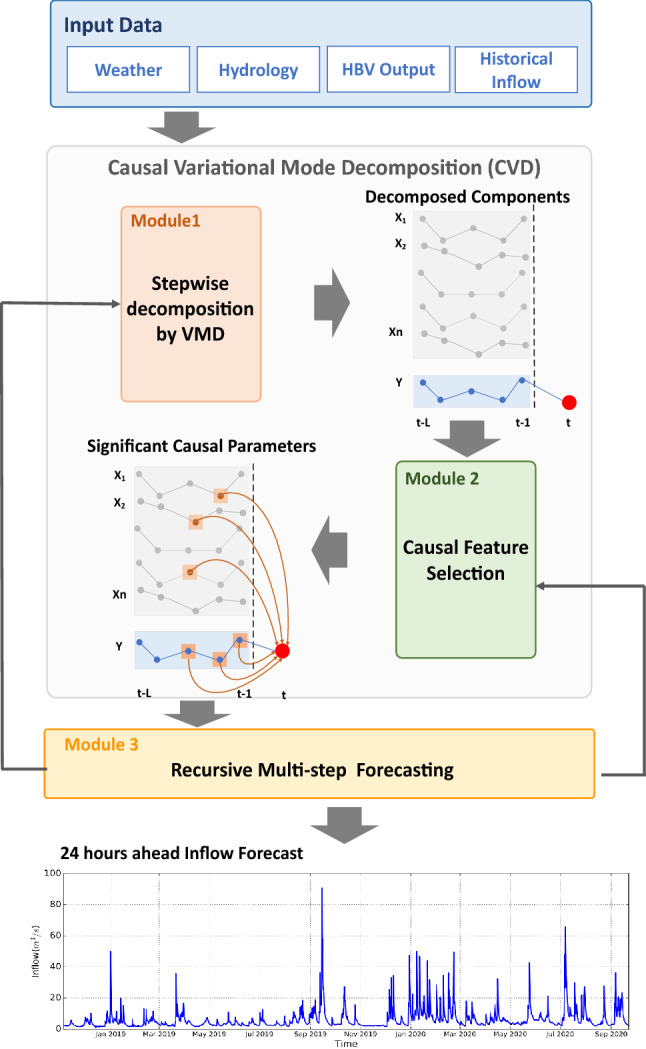
Overview of the proposed CVD framework: Module 1 decomposes the 33 inputs to $n=m*N$ inputs. Module 2 output is the lag values of variables with a maximum contribution to the next step of inflow *Y*. Module 3 output is 24 ahead of inflow forecast by using a one-step ahead trained model.

We collected data in three categories: (1) meteorological, (2) hydrological, and (3) HBV software simulation data^18^. The collected data and locations corresponding to the data are presented in Table 1. The meteorological data includes temperature, and precipitation from Location1 and Location8. The meteorological data is open source and is available on Senorege or Norway klimaservicesenter^19^. Lyse provides the hydrological data for various locations, including water level, water temperature, amount of water dispatch, spillage, and inflow. The last category is simulated hydrological data from the commercial HBV software^18^, which includes average catchment soil water, precipitation, air temperature, evaporation, groundwater, snow water equivalent, and snow melt. The reason for using the HBV model is to use the estimation of variables that have not been measured. Moreover, using the HBV model can indirectly capture the HBV modeling process and catchment physics during training machine learning algorithms. As presented in Table 1, there are two types of inflow provided by HBV: one is the average of inflow for the entire catchment and another is inflow at Location8. The HBV-inflow at Location8 is calculated by temperature, evaporation and precipitation at each time. Moreover, the historical inflow at Location8 in the hydrological category is the target variable *Y* which is highlighted in Table 1. we did not decompose historical inflow to investigate later the dependencies among historical inflow with other input variables and perform a recursive multi-step forecasting technique. Therefore, there are 30 variables in total, but 29 variables are applied to the step-wise sampling decomposition module and inflow *Y* is joined to the output of the decomposition module as presented in Fig. 3. All the data collected for this paper are starting on November 4th, 2018 to the fifth of January 2021 on an hourly basis. For the sake of better visualization, the inflow dispatch at Location1, and Location8 are presented in Fig. 2. As presented, the inflow at Location8 is adjusted by releasing water from Location1 whenever the inflow at Location8 is near a certain threshold value (minimum allowable inflow) to meet the NVE’s environmental requirements.

**Table 1:** Collected data.

Group	Variable	Location	Unit
Meteorological	Air temperature	Location8	$^\circ$C
		Location1 (Briavanet)	$^\circ$C
	Precipitation	Location8	mm
		Location1	mm
Hydrological	Water level	Location2 (Musdalsvatn)	m
		Location3 (Musdalvatn downstream)	m
		Location4 (Viglesdalsvatn)	m
		Location5 (Hiavatn)	m
		Location6 (Hiafossen)	m
		Location7 (Lyngsåna)	m
	Water temperature	Location2	$^\circ$C
		Location3	$^\circ$C
		Location4	$^\circ$C
		Location5	$^\circ$C
		Location6	$^\circ$C
		Location7	$^\circ$C
		Location8	$^\circ$C
	Inflow	Location8	m$^3$/s
		Location7	m$^3$/s
		Dispatch at Location1	m$^3$/s
		Spillage at Location7	m$^3$/s
Simulated hydrological from HBV model	Inflow	Average of catchment	m$^3$/s
		Kalltviet	m$^3$/s
	Evaporation	Average of catchment	mm
	Ground water	Average of catchment	mm
	Soil moisture	Average of catchment	mm
	snow water equivalent	Average of catchment	mm
	Snow melt	Average of catchment	mm
	Precipitation	Average of catchment	mm
	Air temperature	Average of catchment	mm

## Methodology

As explained in the introduction section, the inflow is a multi-stage stochastic problem and is affected by other hydrological variables in a complex way. Therefore, it is essential to use techniques that can reduce the complexities and uncertainties in the inflow forecasting problem. Hence, a Causal Variational Mode Decomposition (CVD) method is proposed as a preprocessing framework in this research. The variational mode decomposition technique reduces the complexity of the time-series by decomposing them to some physically meaningful Modes (features) and causality analysis reduces the uncertainties of inflow by finding lag values of some Modes which have maximum contributions to the next state of inflow. In another word, CVD combines causal inference with multivariate variational mode decomposition to extract the most informative features (Modes) related to the target variable from heterogeneous input data. As shown in Fig. 3, CVD has three main modules. The first module decomposes the input data (meteorological and hydrological data related to the water inflow) into different physically meaningful components called Modes^20^ which is performed by step-wise sampling mechanism^14^. The second module selects the most informative decomposed Modes related to the water inflow with proper time latency values using a greedy search algorithm. In the third module, only the selected latency values of decomposed Modes are used for training a multi-step time-series forecasting algorithm. Each module is further explained in the following sections.

### Step-wise decomposition by VMD

Variational mode decomposition (VMD) is an adaptive non-wavelet multiresolution analysis method that decomposes an original time-series into different Modes without using fixed functions for analysis, similar to EMD^21^. However, in contrast with EMD, VMD is a non-recursive algorithm that extracts *m* Modes (The number of Modes is selected by the user depending on the complexity of the problem) concurrently by identifying all signal peaks in the frequency domain. Therefore, each mode needs to be packed around a center pulsation $\omega _{m}$^21^. For a multivariate time-series $X=[X_{1}(t),X_{2}(t), \dots , X_{N}(t)]$ where *X* is a matrix with size ${N \times T}$, the VMD decomposes each time-series to *m* Modes which results in a new matrix $X_{new}$ with size $n\times T$, $n=N\times m$ subseries. For the sake of better understanding, the VMD formulation is presented for $X_{1}(t)$ as follows:

1 $$\begin{aligned} {\begin{array}{*{20}{c}} {\mathop {\min }\limits _{\{ Mod{e_m}\} ,\{ {\omega _m}\} } \left\{ {\sum \limits _m {\left\| {{\partial _t}\left[ {\left( {\delta \left( t \right) + \frac{j}{{\pi t}}} \right) *Mode{}_m\left( t \right) } \right] {e^{ - j{\omega _m}t}}} \right\| } _2^2} \right\} }\\ {S.t,\,\,\,\,\,\,\,\,\,\sum \limits _m {Mod{e_m}\left( t \right) = {X_1}\left( t \right) \,\,\,} } \end{array}} \end{aligned}$$

where *m* subseries $Mode_{m}(t)$ with the same lenght of *T* are decomposed by VMD. The variable $\omega _{m}$ is the corresponding center frequencies for each Mode. The variable $\delta$ is the Dirac distribution, *t* is time script, and $*$ denotes convolution^21^.The optimization problem in Eq. (1), can be solved by turning it to a Lagrangian form and adding a quadratic penalty term to render the problem unconstrained as follows^22^:

2 $$\begin{aligned}&\mathscr{L}(\{ Mod{e_m}\} ,\{ {\omega _m}\} ,\lambda ): = \alpha \mathop \sum \limits _m \left\| {{\partial _t}[(\delta (t) + \frac{j}{{\pi t}})*Mod{e_m}(t)]{e^{ - j{\omega _m}t}}} \right\| _2^2 + \left\| {{X_1}(t) - \mathop \sum \limits _m Mod{e_m}(t)} \right\| _2^2\nonumber \\&\quad + \langle \lambda (t),{X_1}(t) - \mathop \sum \limits _m Mod{e_m}\rangle \end{aligned}$$

where $\mathscr {L}$ is the Lagrangian function, $\lambda$ is the dual variables, and $\alpha$ indicates the balancing parameter of the data-fidelity constraint. The Alternate Direction Method of Multipliers (ANMM) is employed in VMD to solve the Lagrangian problem presented in Eq. (2). The variables in Eq. 2, such as $\lambda$, $Mode_{m}$ and $\omega _{m}$ are updated as follows^21^:

3 $$\begin{aligned}&\hat{\omega }^{i+1}_{m}=\frac{\int _{0}^{\propto }\omega |\hat{Mode}_m(\omega )|^2 d\omega }{\int _{0}^{\propto }|\hat{Mode}_m(\omega )|^2 d\omega }) \end{aligned}$$

4 $$\begin{aligned}&\hat{\lambda }^{i+1}(\omega )=\hat{\lambda }^n(\omega )+\tau (\hat{X_{1}}(\omega )-\underset{m}{\sum }\hat{Mode}^{n+1}_m(\omega )) \end{aligned}$$

5 $$\begin{aligned}&\hat{Mode}^{i+1}_{m}(t)=Re\mathscr {F}^{-1}\left( \frac{\hat{X_{1}}(\omega )-\underset{i\ne m}{\sum } \hat{Mode}_{i}+\frac{\hat{\lambda }(\omega )}{2}}{1+2\alpha (\omega -\omega _{m})^2}\right) \end{aligned}$$

**Figure 4:**
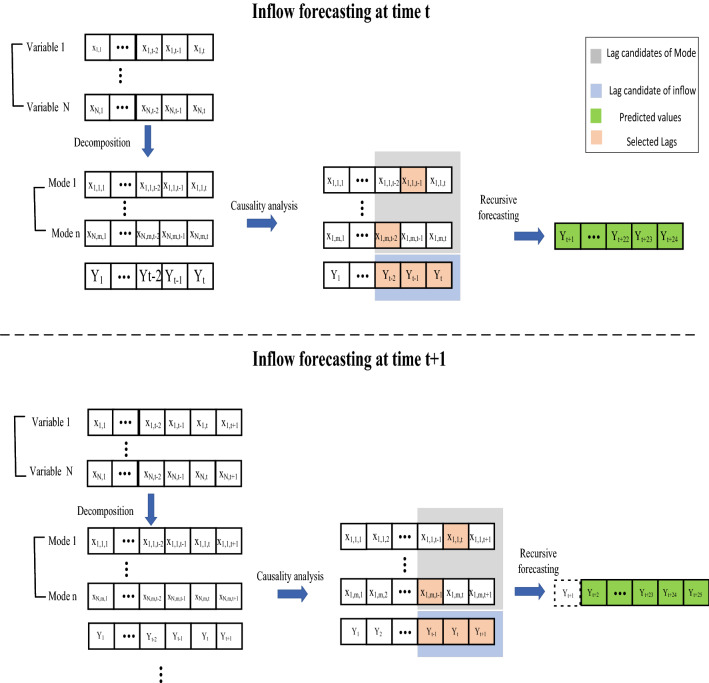
Actual forecasting practice by using Step-wise sampling mechanism for VMD decomposition.

Readers are referred to^21^ for a detailed introduction to VMD. Moreover, the entire time-series is not decomposed at the beginning to avoid any information leakage from the test and validation data to the training dataset. Instead, a step-wise sampling mechanism is used which is presented in Fig. 4. In this mechanism, first, the data is split into training, validation, and test data. Then the entire training time-series is decomposed. The causality inference network is also obtained over the decomposed training samples for training forecast models. Then each sample in the validation or test dataset is appended to the training data set and the VMD decomposition is used to decompose the extended new series for forecasting inflow. This procedure is repeated until all of the samples in the test and validation dataset have been added to the training dataset. This procedure is explained in detail in^14^.

### Causal feature selection algorithm

Our proposed causal feature selection algorithm is obtained based on a greedy search algorithm which maximizes the transfer entropy (TE) from predictor variables in $X_{new}$ which is matrix with size $N\times m\times T$ (Modes of metrological and hydrological variables) to the target variable *Y*(*t*)(inflow at Location8). TE is a model-free metric that measures the direct information transfer from other Modes in $X_{new}$ to the target variable *Y*(*t*). For example, quantifies the information transfer from subseries $X_{1,1}(t)$,$X_{2,m}(t)$, etc., to the target variable *Y*(*t*) where $X_{1,1}(t)$ is Mode1 of original time-series $X_{1}(t)$ or $X_{2,m}(t)$ is Mode m of original time-series $X_{2}(t)$. TE is an extended version of conditional mutual information (CMI). Thereby, the mathematical expressions of CMI and TE for the above mentioned example are presented as follows. The CMI $I(Y(t);X_{2,m}(t)|X_{1,1}(t))$ is^23^:

6 $$\begin{aligned} I(Y;X_{2,m}|X_{1,1})=H(Y|X_{1,1})-H(Y|X_{2,m},X_{1,1}) \end{aligned}$$

7 $$H(Y|X_{1,1})=-\sum _{y\in Y, x_{1,1}\in X_{1,1}}P(y,x_{1})\log _{2} \frac{P(y,x_{1})}{P(x_{1})}$$

where $H(Y|X_{1})$ and $H(Y|X_{2},X_{1})$ are the conditional entropy and $P(x_{1})$ and $P(y,x_{1})$ are the marginal probability of $x_{1}$ and joint probability between *y* and $x_{1}$ realization respectively. The expression of the $TE_{X_{1}\rightarrow Y}$ is as^23^:

8 $$\begin{aligned}&TE_{X_{1,1}\rightarrow Y}=I(Y(t);X_{1,1}[t-1:t-k]|Y[t-1:t-k])\nonumber \\&\quad = H(Y(t)|Y(t-1:t-k))-H(Y(t)|Y(t-1:t-k),X_{1,1}(t-1:t-k)) \end{aligned}$$

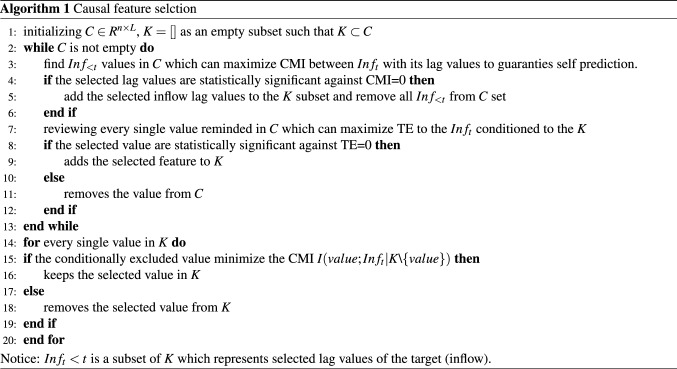


Transfer entropy is the objective function of the greedy search algorithm for causal feature selection. As it is presented in Algorithm 1, there are two main loops in the algorithm. Loop 1 finds the maximum TE between lag values of the inflow and other decomposed Modes using the greedy search method. Loop 2 calculates the causality strength of selected candidates by conditionally removing the selected candidate and evaluating TE for pruning and removing redundant candidates that might have been selected in the first loop.

Therefore, the first step is to initialize the selected subset *K* as an empty set and set *C* as a matrix with size $n\times L$ as the main search space set. Note that $K\subset C$ and *L* is the window size of lag values in inflow and $X_{new}$ (*L* is selected based on the knowledge domain with the assumption that all Modes are Markovian with *L* lags). The variable *d* is the size of *K* subset, representing the number of selected values in the subset of *K* which is zero at the beginning. In the first step, the lag values of Modes that can maximize TE are added to the chosen subset *K*. In the second loop, the selected lag values are checked and pruned by conditionally removing that feature from the subset of *K*. For example, if the TE of the new subset *K* with size $d-1$ is improved, the conditionally removed feature from the *K* subset will be deleted. Otherwise, the conditionally removed feature from the *K* subset will be added. This loop continues as long as all features get tested.

### Recursive multi-step forecasting

In this section, the significant causal set from the previous module is fed to forecasting models to forecast the next 24 h of inflow. We use a recursive multi-step forecasting strategy in this paper to reduce the overall computation time^24^. The recursive multi-step forecasting employs a one-step model multiple times in which the outcome of the first step is used as input for forecasting the second step and so on.

**Figure 5:**
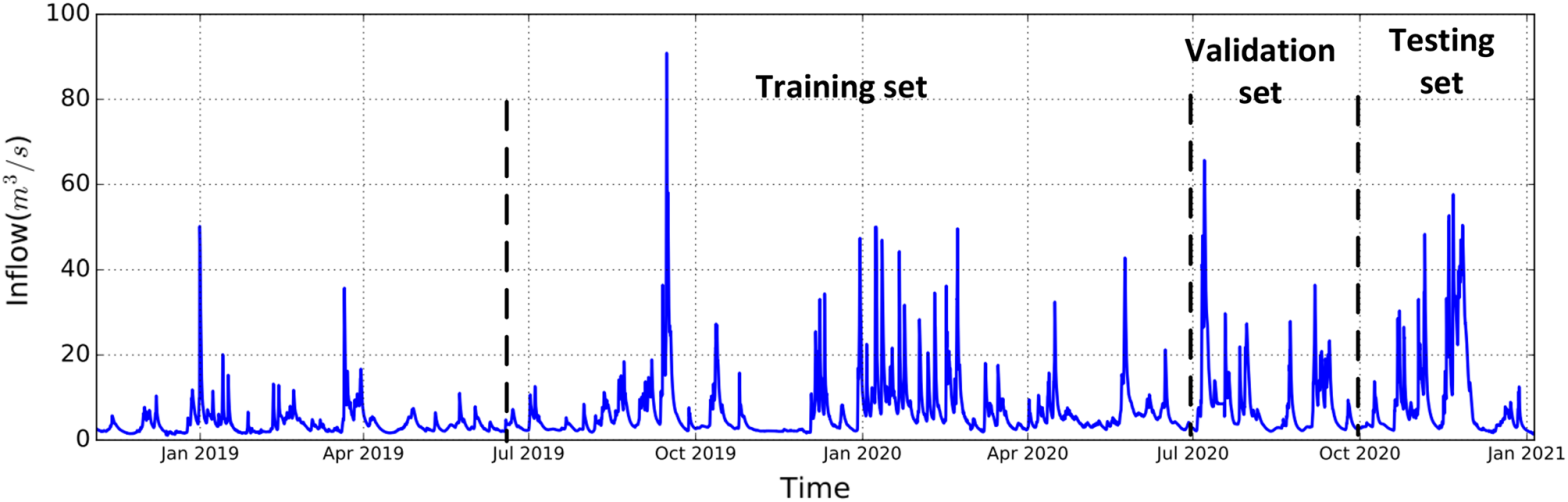
Hourly inflow time-series at Location 8: one year of data is used for training and 6 months of data is used for validation and testing.

In this paper, the CVD framework is tested with four popular forecasting machine learning algorithms: linear regression (LR), random forest regression (RF), multilayer perceptron (MLP), and Long short-term memory (LSTM). These are widely used in the literature for short-term and long-term inflow forecasting. The data in this paper is from November 2018 to January 2021 on an hourly basis. One year of data is used for training and six months of data is used for validation and testing as shown in Fig. 5. In this paper, the data for inflow forecasting is not shuffled, and the last 3 months are used for testing results. For tuning the parameters, a grid search algorithm is used to find the number of hidden layers, number of neurons, optimization method, learning rate, epoch number, and batch size.

## Results

The simulation has been performed on a laptop with Core i7 Intel GPU, 32 GB RAM, and NVIDIA GeForce RTX 2080. In addition, the grid search algorithm is used to find the hyperparameters of each model, like LSTM, MLP, and RF. Then, the results related to each module are presented. We used four well-known performance metrics, Nash-Sutcliffe efficiency (*NSE*), Mean squared error (*MSE*), Normalized Mean squared error and Standard Deviation (*Std*) for the error as follows:

9 $$\begin{aligned}&MSE=\frac{\sum _{t=1}^{T}(Y(t)-\widehat{Y}(t))^2}{T} \end{aligned}$$

10 $$\begin{aligned}&NRMSE=\frac{\sqrt{\frac{1}{T}\sum _{t=1}^{T}(Y(t)-\widehat{Y}(t))^2}}{\overline{\rm{Y}}} \end{aligned}$$

11 $$\begin{aligned}&NSE=1-\frac{\sum _{t=1}^{T}(Y(t)-\widehat{Y}(t))^2}{\sum _{t=1}^{T}(Y(t)-\overline{\rm{Y}})^2} \end{aligned}$$

12 $$\begin{aligned}&Std=\frac{\sqrt{\sum _{t=1}^{T}(e_{t}-\overline{\rm{e}})^2}}{T} \end{aligned}$$

where $\overline{\rm{Y}}$ is the mean of inflow values. The $\widehat{Y}(t)$ is the prediction of inflow, and *Y*(*t*) is the actual value of inflow at time *t*, respectively. Moreover, $e_{t}=Y(t)-\widehat{Y}(t)$ is the error of prediction at time *t* and $\overline{\rm{e}}$ is the average of prediction error. The MSE or NRMSE are recommended for inflow forecasting problems because they penalize larger errors exponentially. It is primarily critical during the snow-melting periods or rainy seasons. We use MSE index to measure the training performance. Our goal is to reach higher accuracy in inflow forecasting, which at a higher level leads to better utilization of water capacity, reduction of reservoir spillage and reduces the environmental and social cost of the floods^25^. NSE is a great metric to assess the predictive skill of hydrological models and in a situation of a perfect model the $NSE=1$. Moreover, Std is used to measure the error forecast probability distribution over forecast values of the entire future horizon.

### Results for variational mode decomposition module

**Figure 6:**
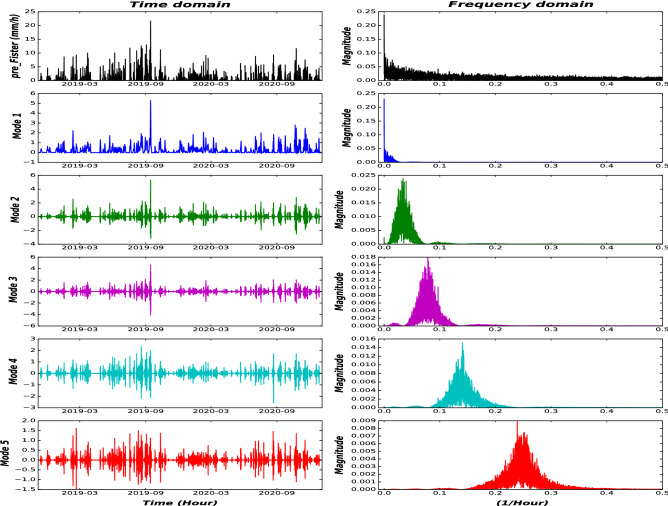
VMD decomposition of precipitation time-series from Fister location.

**Figure 7:**
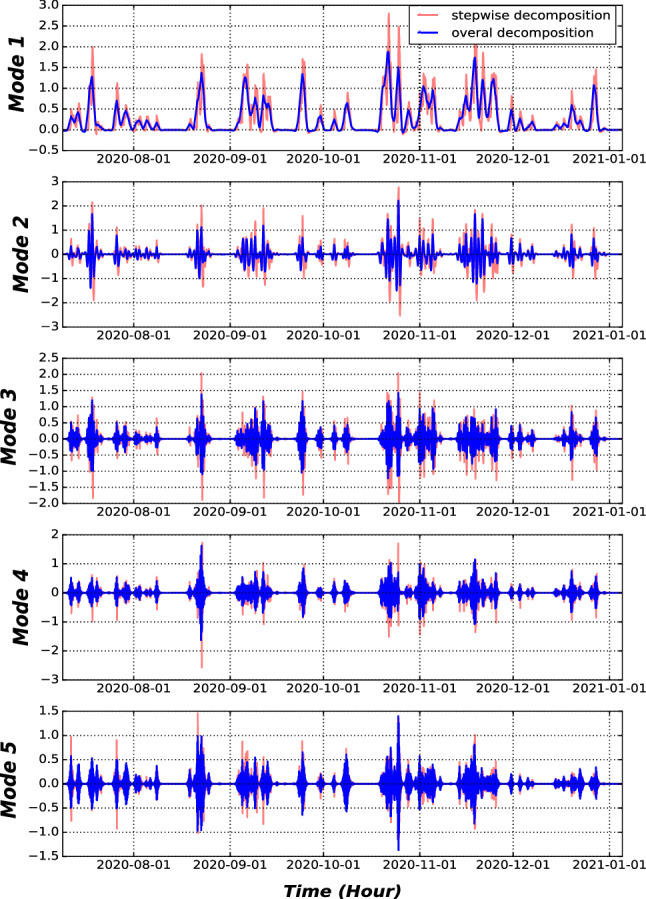
Comparison of VMD decomposition by using the step-wise mechanism or overall-decomposition mechanism on validation and test dataset.

The outcome of the first module is a set of decomposed time-series using the VMD approach with the following parameters: $\alpha =2000,\,\, \tau =0,\,\, m=5,\,\, Dc=0,\,\, init=1$, and $tol=1e^-7$. These parameters were chosen heuristically based on the work on^23^. We set the number of Modes to 5 by trial and error to avoid the curse of dimensionality and computational complexity. We have 29 time-series variables, each with 5 Modes, which amounts to 145 Modes (subseries) plus inflow time-series, for a total of 146 time-sereis with a length of training samples. For the sake of better visualization, we show the decomposition results of precipitation at Location8 (as presented in Fig. 1) in Fig. 6 by using the step-wise decomposition mechanism. Moreover, the decomposition by using a step-wise mechanism is compared with the overall decomposition mechanism in Fig. 7. As it is presented in Fig. 6, the Mode 1 represent low frequencies (monthly and seasonality patterns of precipitation), while Mode 5 consists of the highest frequencies that represent hourly changes in the precipitation. In addition, in Fig. 7, the Modes acquired by Step-wise decomposition are higher and have more oscillations compared with the overall decomposition-based mechanism because the overall decomposition mechanism uses future information to have a more accurate estimation of upper and lower envelopes^14^.

**Figure 8:**
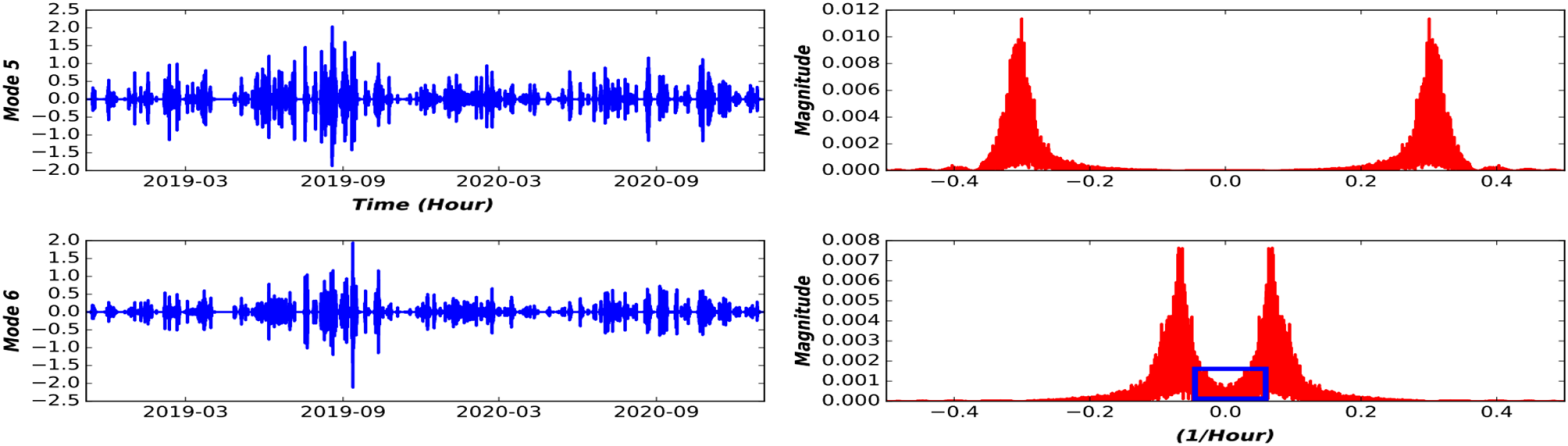
Center frequency aliasing of the last Mode.

The next step is to create a causal model out of decomposed time-series (165 Modes in our case) to keep the most informative Modes regarding the target variable and avoid redundancy. In addition, the causal Modes down-selecting reduces the computing time and makes practical implementation easier. As mentioned earlier, the number of Mode is set to 5, however, it is a difficult task to find the number of Modes that the original series should be decomposed into. Too many Modes will be computationally expensive and intensive for both causality analysis and training whereas few Modes may not properly extract features inside raw data. The number of Modes in this paper is found by the experiment of the obvious aliasing phenomenon of the center frequency for the last component. When the $m= 6$, the frequency spectrum of the 6th Mode has obvious aliasing phenomena (area surrounded by a blue rectangular border shown in Fig. 8) which is presented in Fig. 8. Hence, *m* is set to 5 to make the decomposition result satisfy orthogonality and avoid high computational complexity and curse of dimensionality problem for causal analysis.

### Causal feature selection results

We perform the causal feature selection process to find the most informative Modes which have the most contribution to the next state of the target variable. In this paper, the target variable is the water inflow at Location8, which is the inlet to the Fjord.

The causal inference reduces the decomposed Modes (with different time lags) set’s size from 146 to a smaller size. We assume the values per Mode are Markovian by 24-h lag values (representing one full day, $L=24$). It is not possible to consider all the lag values because it is an NP-hard problem that is not tractable. Changing this Markovian assumption to longer or shorter lag intervals may result in different causality sets. However, in this paper, we are trying to show that with these 24-h Markovian conditions, we can find a fair forecasting accuracy while trying to limit computational complexity. Then, using the Transfer Entropy (TE) metric, the causal feature selection algorithm searches among 3504 lag values $(146\times 24 = 3504)$. The causal inference results show that, the target value, *Y*(*t*), is primarily dependent to the five first lags of itself $Y(t-1),\,\,\, Y(t-2),\,\,\,Y(t-3)$, $Y(t-4)$ and $Y(t-5)$ as well as specific time lag of other decomposed components (Modes) as presented in Table 2.

**Table 2:** Significant selected Modes by causal feature selection algorithm.

Variable	Location	Mode	Lag
Precipitation	Location8 actual (Kalltviet)	3	4
	Average of catchment HBV	3	3
		5	4
Inflow	Location7 actual (Lyngsåna)	2	6
		3	1
		4	12
		5	12
	Location1 actual (dispatch)	3	12
	Average of catchment HBV	3	12
		4	1
		5	12
	Location8 HBV	1	1
		2	1
		3	5
		4	2
		5	2
Ground water	Average of catchment HBV	4	3
Water level	Location3 actual (Musdalsvatn down stream)	2	2
		5	1
	Location7 actual	1	1
		3	1
	Location6 actual (Hiafossen)	3	5
		4	7
		5	2
	Location5 actual (Hiavatn)	3	12
		4	1
		5	2
	Location2 actual (Musdalsvatn)	3	1

**Figure 9:**
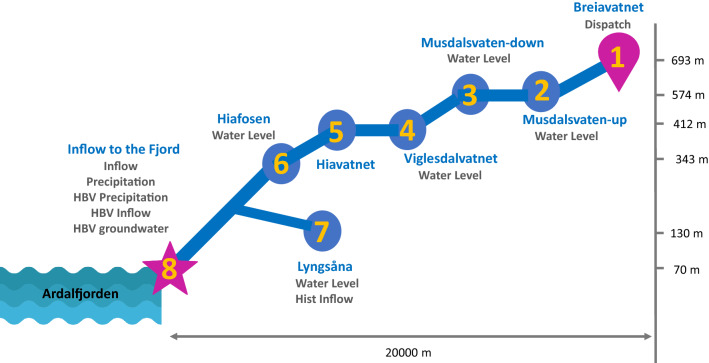
Geo-spatial relationship between selected causal candidates by using the CVD.

The outcome of the causal inference inherits the underlying governing dynamics between various hydrological and meteorological parameters (either actual measurement or HBV simulation data) through extracting cause and effect relationships between input decomposed variables as shown in Table 2 from the dispatch point at lake Breiavatnet (Location1) at 693 m altitude to the Ardal fjord’s inlet point at downstream and 20 km away. Figure 9 shows that the inlet to the Ardal Fjord is highly dependent on measurement at the same point, with time lags varying from 5 h ago to the last hour. Another interesting observation is that the water levels in upstream Locations 1–7 are essential parameters that can impact the water inflow at the inlet point (Location8). Moreover, the available meteorological data are only for Locations1 and 8. Not surprisingly, the meteorological parameters of Location8 significantly impact the inflow at the exact location rather than the dispatch point (Location1) 20 km up the mountain. In addition, it is shown that dispatch water from Location1 with a lag of 12 h has an impact on inflow at Location8 which can be interpreted as the time it took to see the effect of dispatch on inflow at Location8. Therefore, it is very important for our use case to have forecasting models to accurately forecast 12 to 24 h ahead of inflow to minimize the water dispatch from Location1. It is exactly what the Lyse production AS is doing to look at the forecast of inflow from $t+12$ to $t+24$ to decide how much water they should release from Location1.

As you can see in Table 2, only 28 values from Modes and 5 values from inflow lags 33 values in total are selected using our proposed causal feature selection among 3504 values to make the causal set for the hourly inflow forecasting.

### Multi-step forecasting results

In this section, four different models are trained using CVD preprocessing modules to forecast 24 h ahead of inflow at the measurement point (Location8). But before starting to train each model, it is necessary to perform normalization and some sensitivity analysis regarding the sub-optimal hyperparameters and size of historical horizon data.

#### Normalization

Since the range of inflow and other Modes decomposed from meteorological and hydrological data vary widely, it is necessary to normalize all input data^22^. In this paper, all the variables are normalized to the same scale by using the MinMaxScaler package from scikit-learn^26^ from range $-1$ to 1. The normalization formula is presented for variable $X_{1,1}(t)$ as follows:

13 $$\begin{aligned} X_{1,1,Normalized}(t)=\frac{X_{1,1}(t)-X_{1,1,min}}{(X_{1,1,max}-X_{1,1,min})} \end{aligned}$$

where $X_{1,1,Normalized}(t)$ is the normalized vector which is calculated by element-wise mathematical operations. The variables $X_{1,1}(t)$, $X_{1,1_min}$ and $X_{1,1,max}$ are the Mode 1 of first variable in vector *X*(*t*) and its corresponding minimum and maximum values, respectively. The normalization method is only fitted on training data and used for scaling test and validation data later to avoid leakage information from validation and test data.

#### Sensitivity analysis

**Figure 10:**
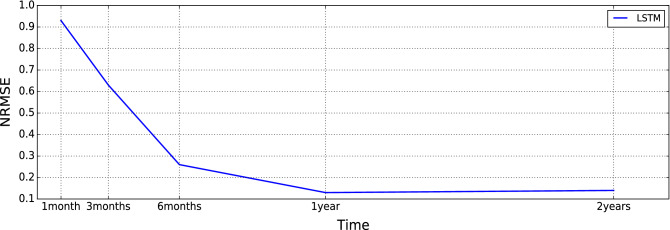
Sensitivity analysis on different training horizons (notice the loss error here is for one hour ahead forecast model of a 1-month test dataset).

Since LSTM is widely used in time-series forecasting applications, including inflow, for performing sensitivity analysis, an LSTM with historical weather and inflow data time-series is trained^27,28^. First, we use a grid search algorithm to find out the hyperparameters of an LSTM to forecast one step ahead of inflow. Per our investigation, LSTM shows a better performance for inflow forecast in our use case. Later, we will explain it in more detail. The sub-optimal values for LSTM hyperparameters are presented in Table 3. For evaluating the size of training data, a sensitivity analysis has been done for different ranges of training horizons [2 years, 1 year, 6 months, 3 months, and 1 month] to find the sub-optimal length of training size of data. The LSTM performance for different training sizes is presented in Fig. 10, based on NRMSE criteria for the next hour.

**Table 3:** LSTM hyperparameters.

Activation function	Relu	Learning rate	0.001
Number of hidden layers	2	Optimizer	Adam
Loss function	MSE	Epoch	100
First layers neurons	50	Neurons of second hidden layer	500
Batch size	64	Neurons of output layer	1

As it is presented, increasing the training size to one-year data can significantly improve the loss error from 0.9 to 0.13. After a 1-year duration, the improvement in error reaches a saturation zone, in contrast.

It is essential to train the model adequately to perform accurate one-step ahead forecasting because the same model will be used recursively to forecast the entire future horizon. So, we limit the error propagation.

From a practical point of view, the computational time for training models shall be fast for short-term hydropower scheduling problems. Therefore, in this paper, all the models are trained based on 1-year data, considering the trade-off between computation time and accuracy.

Since LSTM, MLP, LR, and RF are well-known forecasting methods for multi-variate inflow forecasting, we add CVD as a preprocessing step before all of them. Table 4 presents the inflow forecast in Location8. The error values are the average day-ahead forecast error over 90 sequential days (mid of October 2020 to the mid of January 2021). As presented in Table 4, the CVD-LSTM outperforms all the other forecast algorithms for forecasting inflow for 24-h ahead. As an observation, all the models have demonstrated excellent performance for forecasting one to six hours ahead of inflow but the error propagation is increased after $t+6$ while CVD-LSTM has less propagation in error by $t+24$ compared with others in terms of all provided metrics in this paper.

**Table 4:** Comparison of using CVD with different forecasting models.

Models	Metrics	Future forecast horizons					
		t + 1	t + 2	t + 6	t + 12	t + 18	t + 24
CVD-RF	NRMSE	0.08	0.13	0.28	0.49	0.57	0.68
	NSE	0.98	0.98	0.93	0.8	0.73	0.61
	Std	0.9	1.27	2.71	4.71	5.53	6.63
CVD-LR	MSE	0.06	0.1	0.28	0.41	0.49	0.55
	NSE	0.99	0.99	0.93	0.85	0.79	0.75
	Std	0.7	1.02	2.65	4.02	4.8	5.27
CVD-MLP	MSE	0.12	0.17	0.32	0.41	0.51	0.53
	NSE	0.97	0.97	0.91	0.86	0.78	0.77
	Std	1.2	1.66	3	3.9	4,89	5.09
CVD-LSTM	MSE	0.1	0.16	0.31	0.38	0.46	0.51
	NSE	0.98	0.97	0.92	0.88	0.84	0.8
	Std	0.8	1.7	3.1	3.8	4.04	4.9

To evaluate the role of CVD as a pre-proccessing feature selection framework in improving the LSTM forecasting performance, new results have been presented in Table 5. The forecast performance, NRMSE and computational time is compared with a stand-alone LSTM for four different scenarios. Only historical inflow are used for scenario 1, weather data are used for scenario 2, weather & hydrology are used for scenario 3, and weather & hydrology & HBV data are used for scenario 4.

There are three interesting observations in Table 5.

- The first observation is that by comparing only LSTM with CVD-LSTM across four scenarios, the NRMSE error reduces significantly. For example, there is a 70% reduction in the error at scenario 4 due to CVD integration into LSTM compared with scenario 1. Moreover, we have 25% improvement inside scenario 4 when CVD is added to LSTM.
- The second observation is that the error is reduced by adding more input variables from scenario 1 (historical inflow only) to scenario 4 (weather + hydrology + HBV). For example, the CVD-LSTM error in scenario 4 is 50% less than in scenario 2.
- The last observation is the computation time reduction. Adding hydrological and HBV data improves LSTM forecast performance whether CVD is used or not. However, without CVD it results in increasing computational time. For example, in scenario 4 the computational time for CVD-LSTM is around 76 (s), which is almost similar to scenario 1, while without CVD it is 900 (S). Hence, The CVD framework can be beneficial for inflow forecasting problems because it will save a considerable amount of computational time and also reduce error.

**Table 5:** Comparison of input data impact on LSTM and CVD-LSTM performance.

Senarios	Data	Model	period	NRMSE	Computational time (s)
1	Historic inflow	LSTM	t + 24	1.7	547
2	Weather	LSTM	t + 24	1.66	442
		CVD-LSTM		1.03	80
3	Weather + hydrological data	LSTM	t + 24	1.06	629
		CVD-LSTM		0.8	96
4	Weather + hydrological + HBV data	LSTM	t + 24	0.68	900
		CVD-LSTM		**0.51**	**76**

Significant values are in bold.

**Figure 11:**
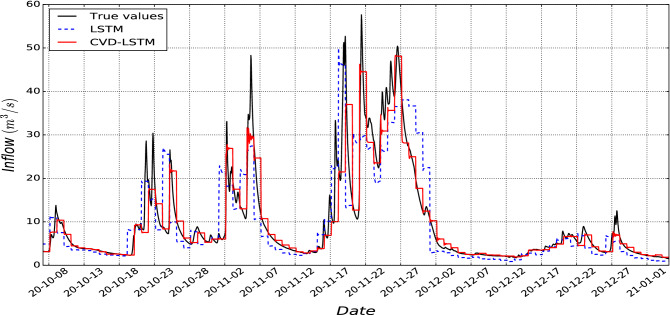
An example of inflow forecasting with CVD-LSTM and LSTM compared with true values for scenario 4.

For visualization purposes, we show CVD-LSTM and LSTM forecasting results in comparison to actual values of the inflow for the test dataset (October 2020 to January 2021) in Fig. 11 for scenario 4. It is clear that the CVD-LSTM better matches the true values rather than only LSTM for 24-h ahead forecasting.

Finally, we analyze the CVD-LSTM *model performance drift* to find a proper retraining interval that maintains the model performance. Since the model is trained based on one year of historical data, the model forecast performance degrades as we move further into the future. Therefore, regular retraining of the model is essential as updated data will be available.

**Figure 12:**
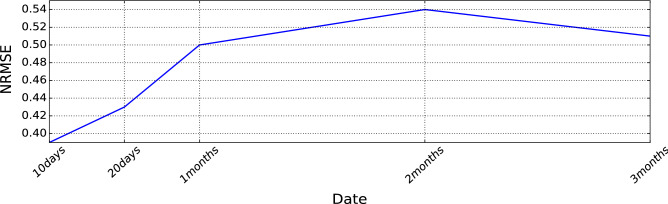
Model performance drift over different sequential day-ahead forecasting periods.

We test our trained model for various future horizons [10 days, 20 days, 1 month, 2 months and 3 months] and measure the performance drift considering our available data from the data provider (Lyse company). As it is presented in Fig. 12, From one month to three months, there is a drift in the model performance in which the loss error is increased by 25%. However, for three months of data, the model performance improve a Little because there was less oscillation in month 3 compared with month 2.

Therefore, we suggest retraining the model between 20 to three-month intervals for our use case. Hence, the retraining interval may vary case by case.

## Practical consideration for hydro power producers

The water regime in a river is dynamic due to seasonal patterns of water availability depending on precipitation, evaporation, drainage, and other characteristics, which all depend on the geography and weather characteristics of a location. These regimes need to be considered for the strategic usage of the water stored in reservoirs. The patterns and uncertainties associated with water regimes, water inflows into reservoirs and other renewable energy sources production have an important impact on hydropower generation and reservoir storage operation. On the one hand, the accurate inflow forecast ahead of natural disasters avoids unnecessary water abandonment and even substantial economic losses. But on the other hand, it is essential to protect the wildlife and ecology of rivers on normal days.

To our knowledge, the following are some of the practical challenges for accurate inflow forecasts for hydropower producers:

- Current inflow prediction methods adopted by most water managers are based on a “past-to-future” approach that averages and extrapolates historical data to the future. However, historical data are not representative of the current climate change situation.
- Inflow is influenced by various heterogeneous (ecological, meteorological, topographical, etc. ) factors with high interdependencies, especially in cascaded reservoirs. Therefore, it is challenging to select appropriate features for inflow forecasting.
- Lack of the details of operational rules and guidelines makes the scientific community and developers remain uniform mainly on several key elements to develop new tools for water management.
- Computation time for new developed forecasting models should be as less as possible because integrating renewable resources such as wind and solar energy into the grid introduce more uncertainties to the grid electricity market which requires hydropower producers to reduce short-term scheduling from hourly to minute basis intervals.

## Conclusion

This paper proposes a Causal Variational Mode Decomposition (CVD) preprocessing framework for the multi-step ahead inflow forecasting problem. In other words, CVD is a preprocessing feature selection framework for multi-variate time-series, which is built upon multiresolution analysis and causal inference. The CVD can reduce the computation time while increasing the forecasting accuracy by down-selecting the most relevant features to the target value (inflow in a specific location). Moreover, the proposed CVD framework is a complementary step to any machine learning-based forecasting method as it is used four different forecasting algorithms in this paper. We validated the CVD using actual data from a river system downstream of a hydropower reservoir in the southwest of Norway provided by Lyse company, one of the largest electricity producers in Norway. The experimental results prove that using CVD-LSTM improves the day-ahead forecasting accuracy by almost 25% and 70% for different scenarios. In principle, the developed framework can be applied to any other cascaded water system. For future work, we will investigate the validation of the proposed method using other cascaded water systems and we will investigate other possible variables and other causal inference methods.

## Data Availability

The data that support the findings of this study are not publicly available because they are provided by the private section Lyse Produksjon which was used under license for the current study. Data are however available from the authors upon reasonable request and with permission of Lyse Produksjon.
